# Supplementary material for: 6-Shogaol Exhibits a Promoting Effect with Tax via Binding HSP60 in Non-Small-Cell Lung Cancer
Source: Cells. 2022 Nov 19;11(22):3678. doi: 10.3390/cells11223678 (PMC9688423; doi:10.3390/cells11223678)
Supplement: Supplementary file 1 [file cells-11-03678-s001.zip › cells-1994604-supplementary.pdf]

# 6-Shogaol exhibits a promoting effect with Tax via binding HSP60 in non-small cell lung cancerTitle

**Shulipan·Mulati, Rongsong Jiang, Jinfeng Wang, Yicun Tao * and Weiyi Zhang ***

School of Pharmacy, Xinjiang Medical University, Urumchi, 830017, China

* Correspondence: taoyicun@xjmu.edu.cn and zwy@xjmu.edu.cn; Tel.: +86-0991-4362505

In this section, we have provided all the original blots. Each experiment was repeated three times. We have marked the molecular weight markers on the Western blotting images in the final figures of the manuscript. Image software was used for all sensitivity analyses, and we adopt the relative quantitative statistics and normalized the value.

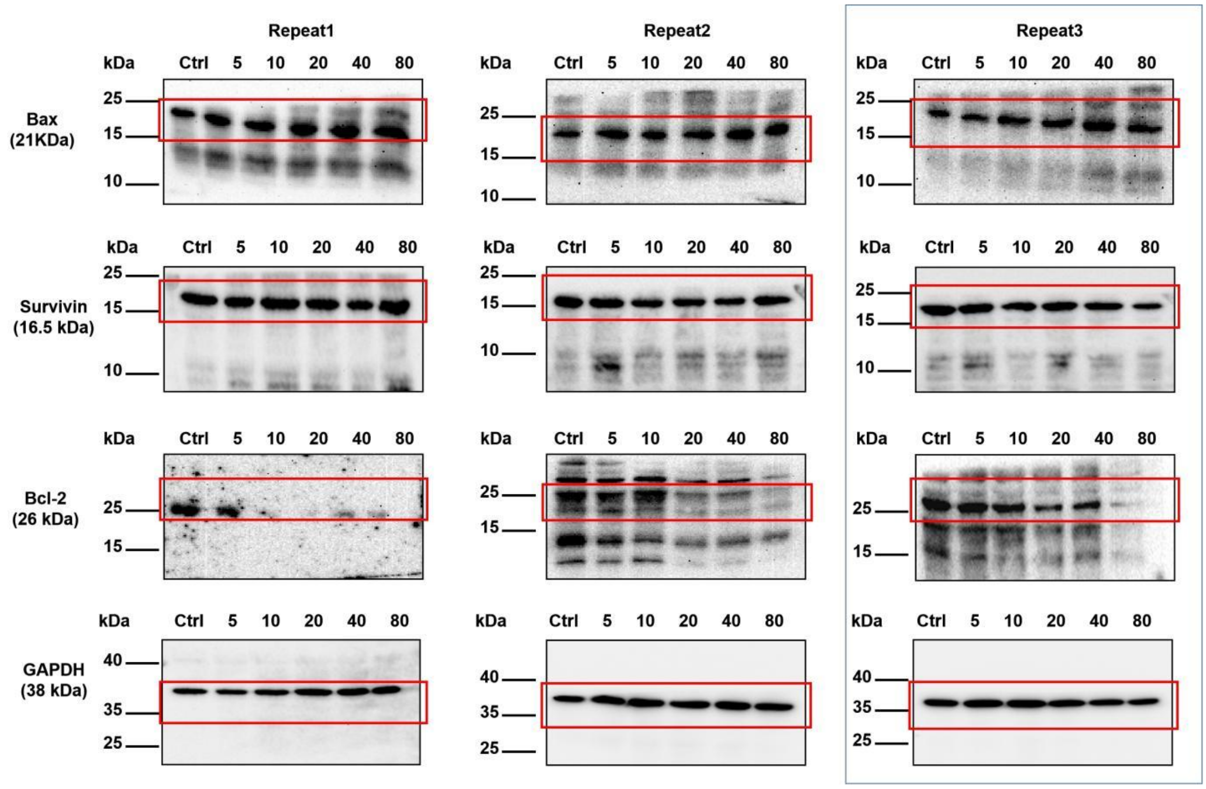

| | | Repeat 1 | | | Repeat 2 | | | Repeat 3 | | | | |
|---|---|---|---|---|---|---|---|---|---|---|---|---|
| Bax | 6-SH (μM) | intden | Normalization to Actin (with same group) | Quadratic normalization to 0 (in each group) | intden | Normalization to Actin (with same group) | Quadratic normalization to 0 (in each group) | intden | Normalization to Actin (with same group) | Quadratic normalization to 0 (in each group) | The mean value of the three normalized groups | The standard deviation of the three normalized groups |
| | Ctrl | 20598 | 0.608759901 | 1 | 27408 | 1.058305661 | 1 | 21988 | 0.70222279 | 1 | 1 | 0 |
| | 5 | 24636 | 0.634114952 | 1.041650331 | 32211 | 0.977512746 | 0.923658242 | 23059 | 0.831854257 | 1.184601623 | 1.049970065 | 0.130670485 |
| | 10 | 26984 | 0.692963534 | 1.13831994 | 33901 | 0.935329011 | 0.883798552 | 26940 | 0.814808094 | 1.160327043 | 1.060815179 | 0.153695291 |
| | 20 | 26583 | 0.784668516 | 1.288962226 | 32533 | 0.963683758 | 0.91059114 | 27735 | 0.727742646 | 1.036341538 | 1.078631635 | 0.192697973 |
| | 40 | 25752 | 0.813623582 | 1.336526242 | 33327 | 0.929934706 | 0.878701438 | 28557 | 0.787475182 | 1.121403625 | 1.112210435 | 0.22905081 |
| | 80 | 26233 | 0.914774907 | 1.502685879 | 29960 | 0.912385419 | 0.862119001 | 25526 | 0.744827989 | 1.060671911 | 1.141825597 | 0.32790383 |
| Survivin | | | | | | | | | | | | |
| | Ctrl | 41877 | 1.237646294 | 1 | 41440 | 1.600123562 | 1 | 45579 | 1.45564001 | 1 | 1 | 0 |
| | 5 | 35602 | 0.916372809 | 0.74041575 | 41926 | 1.272335518 | 0.795148293 | 43639 | 1.574278499 | 1.08150263 | 0.872355558 | 0.183182385 |
| | 10 | 43241 | 1.110451977 | 0.897228863 | 39407 | 1.087239619 | 0.679472289 | 39824 | 1.204488401 | 0.827463104 | 0.801388085 | 0.111195375 |
| | 20 | 40685 | 1.200926855 | 0.970331234 | 35878 | 1.062768447 | 0.664178987 | 39892 | 1.046731915 | 0.719087073 | 0.784532431 | 0.1632318 |
| | 40 | 38853 | 1.227544153 | 0.991837619 | 34463 | 0.961632904 | 0.600974154 | 38884 | 1.072247959 | 0.736616163 | 0.776475979 | 0.198456962 |
| | 80 | 37887 | 1.321163302 | 1.067480514 | 36587 | 1.114200445 | 0.696321504 | 24689 | 0.720405007 | 0.494906022 | 0.75290268 | 0.290450442 |
| Bcl-2 | | | | | | | | | | | | |
| | Ctrl | 54998 | 1.625428538 | 1 | 37175 | 1.989651711 | 1 | 38764 | 1.237991824 | 1 | 1 | 0 |
| | 5 | 42366 | 1.090473862 | 0.670883915 | 29251 | 1.768420733 | 0.888809194 | 36921 | 1.331926407 | 1.075876578 | 0.878523229 | 0.202692168 |
| | 10 | 17771 | 0.456368772 | 0.280768279 | 33155 | 0.745537315 | 0.374707448 | 33506 | 1.013398663 | 0.818582678 | 0.491352802 | 0.287255437 |
| | 20 | 13788 | 0.406989787 | 0.250389222 | 23901 | 0.868775734 | 0.436647142 | 22725 | 0.596284537 | 0.481654665 | 0.389563676 | 0.122611443 |
| | 40 | 21705 | 0.685760324 | 0.421895093 | 28092 | 0.679585914 | 0.341560239 | 26983 | 0.744071255 | 0.601030831 | 0.454828721 | 0.13283341 |
| | 80 | 17121 | 0.597028978 | 0.367305584 | 15300 | 0.99135122 | 0.498253646 | 8450 | 0.24656415 | 0.199164603 | 0.354907944 | 0.14992945 |
| GAPDH | | | | | | | | | | | | |
| | Ctrl | 33836 | 1 | | 25898 | 1 | | 31312 | 1 | | | |
| | 5 | 38851 | 1 | | 32952 | 1 | | 27720 | 1 | | | |
| | 10 | 38940 | 1 | | 36245 | 1 | | 33063 | 1 | | | |
| | 20 | 33878 | 1 | | 33759 | 1 | | 38111 | 1 | | | |
| | 40 | 31651 | 1 | | 35838 | 1 | | 36264 | 1 | | | |
| | 80 | 28677 | 1 | | 32837 | 1 | | 34271 | 1 | | | |

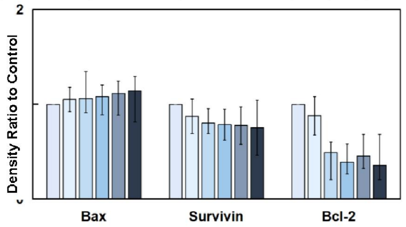


**Supplement Figure S1** Original unedited bands for evaluating 6-SH treatment (0-80 μM) affected apoptotic proteins measured by western blotting in Fig. 2D. Image software was used for sensitivity analysis, and the relative quantitative statistics and normalized the intensity value against "Ctrl" group were adopted. Western blotting was performed 3 times. The "Repeat 3" was used in the manuscript.

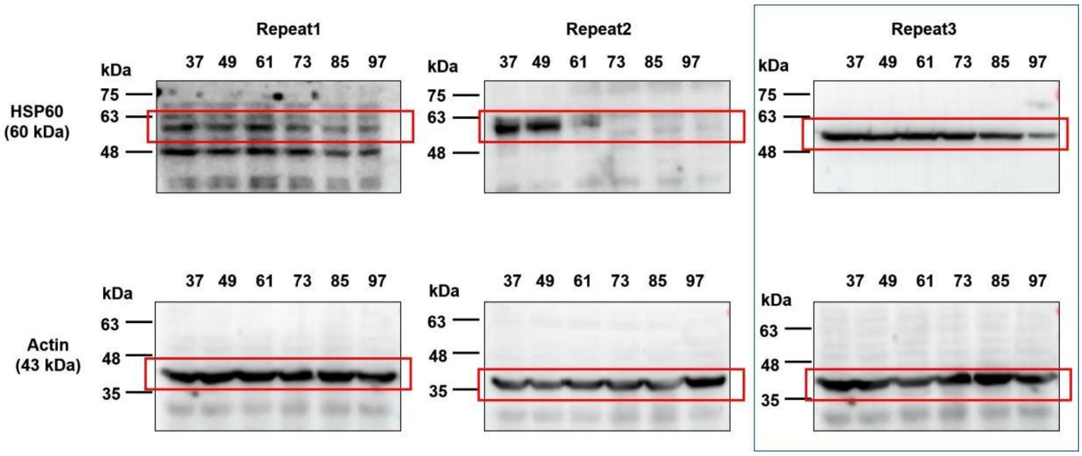

| Repeat 1 | | | Repeat 2 | | | Repeat 3 | | | | |
|---|---|---|---|---|---|---|---|---|---|---|
| intden | Normalization to Actin (with same group) | Quadratic normalization to 0 (in each group) | intden | Normalization to Actin (with same group) | Quadratic normalization to 0 (in each group) | intden | Normalization to Actin (with same group) | Quadratic normalization to 0 (in each group) | The mean value of the three normalized groups | The standard deviation of the three normalized groups |
| 148863 | 1.509567704 | 1 | 125947 | 1.461356385 | 1 | 56800 | 0.562326129 | 1 | 1 | 0 |
| 139586 | 1.503058104 | 0.995687772 | 118451 | 1.243175449 | 0.850699708 | 47940 | 0.459657702 | 0.817421915 | 0.887936465 | 0.094787188 |
| 136572 | 1.430717496 | 0.947766365 | 58848 | 0.617250024 | 0.422381583 | 46719 | 0.467213361 | 0.830858351 | 0.733668767 | 0.275847156 |
| 135448 | 1.492391939 | 0.988622064 | 27313 | 0.320993313 | 0.219654368 | 38264 | 0.363291115 | 0.646050568 | 0.618109 | 0.385244569 |
| 115521 | 1.296677517 | 0.858972747 | 32122 | 0.339380236 | 0.232236462 | 31035 | 0.25912814 | 0.460814689 | 0.517341299 | 0.317168781 |
| 49950 | 0.540397265 | 0.35798147 | 21856 | 0.022255509 | 0.015229351 | 29174 | 0.302095846 | 0.537225339 | 0.30347872 | 0.26523172 |
| | | | | | | | | | | |
| 98613 | 1 | | 86185 | 1 | | 101009 | 1 | | | |
| 92868 | 1 | | 95281 | 1 | | 104295 | 1 | | | |
| 95457 | 1 | | 95339 | 1 | | 99995 | 1 | | | |
| 90759 | 1 | | 85089 | 1 | | 105326 | 1 | | | |
| 89090 | 1 | | 94649 | 1 | | 119767 | 1 | | | |
| 92432 | 1 | | 982049 | 1 | | 96572 | 1 | | | |

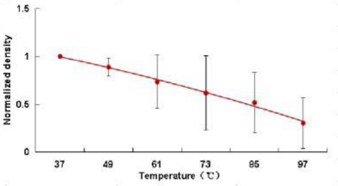


**Supplement Figure S2** Original unedited bands for evaluating 6-SH treatment (20 μM) affected the HSP60 in different Temp. by western blotting in Fig. 4D. Image software was used for sensitivity analysis, and the relative quantitative statistics and normalized the intensity value against “37” group were adopted. Western blotting was performed 3 times. The “Repeat 3” was used in the manuscript.

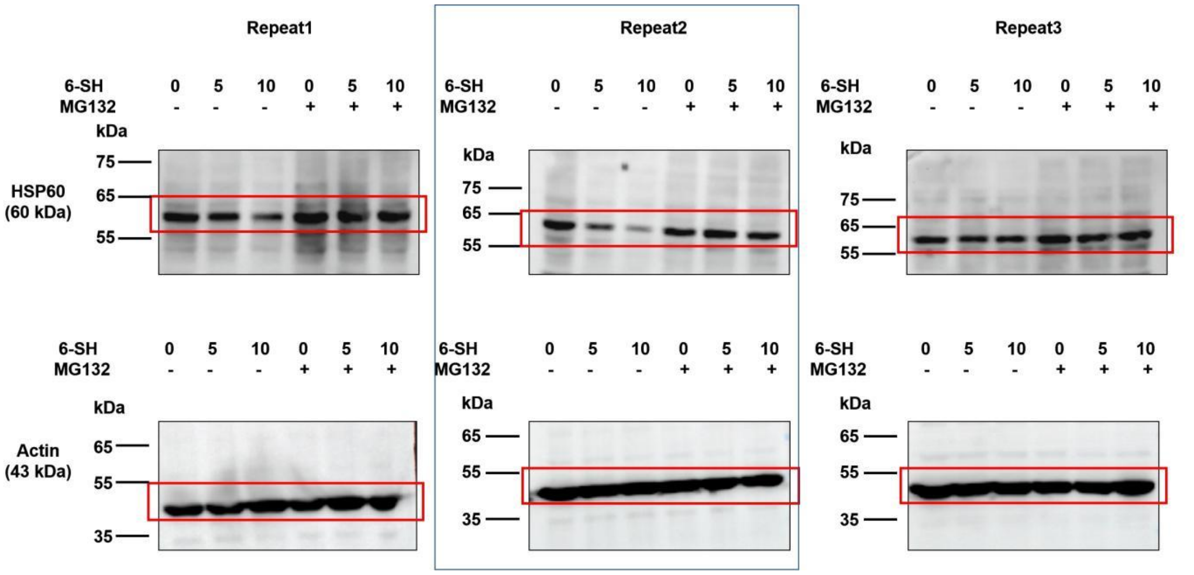


| HSP60 | 6-SH (μ M) | Repeat 1 intden | Normalization to Actin (with same group) | Quadratic normalization to 0 (in each group) | Repeat 2 intden | Normalization to Actin (with same group) | Quadratic normalization to 0 (in each group) | Repeat 3 intden | Normalization to Actin (with same group) | Quadratic normalization to 0 (in each group) | The mean value of the three normalized groups | The standard deviation of the three normalized groups |
|---|---|---|---|---|---|---|---|---|---|---|---|---|
| | Ctrl | 79099 | 0.351178082 | 1 | 79692 | 0.257011275 | 1 | 122703 | 0.477638724 | 1 | 1 | 0 |
| | 5 | 62672 | 0.301950789 | 0.859822423 | 46117 | 0.149208773 | 0.580553415 | 80291 | 0.303619643 | 0.635667979 | 0.692014606 | 0.147915522 |
| | 10 | 47175 | 0.208198176 | 0.592856406 | 28847 | 0.091493836 | 0.355991526 | 55162 | 0.211558596 | 0.442925973 | 0.463924635 | 0.119820494 |
| | Ctrl+MG13: | 68262 | 0.300180736 | 0.854782094 | 68591 | 0.226553133 | 0.881491029 | 108936 | 0.440542388 | 0.922333901 | 0.886202341 | 0.034021449 |
| | 5+MG132 | 74408 | 0.334143154 | 0.951492053 | 78873 | 0.268284636 | 1.043863293 | 119085 | 0.478103245 | 1.000972536 | 0.99877596 | 0.046224779 |
| | 10+MG132 | 77678 | 0.347493491 | 0.989507913 | 76732 | 0.258709688 | 1.006608323 | 118748 | 0.490169612 | 1.026235075 | 1.007450437 | 0.018378057 |
| Actin | | | | | | | | | | | | |
| | Ctrl | 225239 | | | 310072 | | | 256895 | | | | |
| | 5 | 207557 | | | 309077 | | | 264446 | | | | |
| | 10 | 226587 | | | 315289 | | | 260741 | | | | |
| | Ctrl+MG13: | 227403 | | | 302759 | | | 247277 | | | | |
| | 5+MG132 | 222683 | | | 293990 | | | 249078 | | | | |
| | 10+MG132 | 223538 | | | 296595 | | | 242259 | | | | |

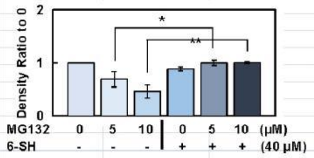


**Supplement Figure S**3 Original unedited bands for evaluating the expression levels of HSP60 after

treating with MG132 by western blotting in Fig. 4G. Image software was used for sensitivity analysis, and the relative quantitative statistics and normalized the intensity value against first group were adopted. Western blotting was performed 3 times. The “Repeat 2” was used in the manuscript.

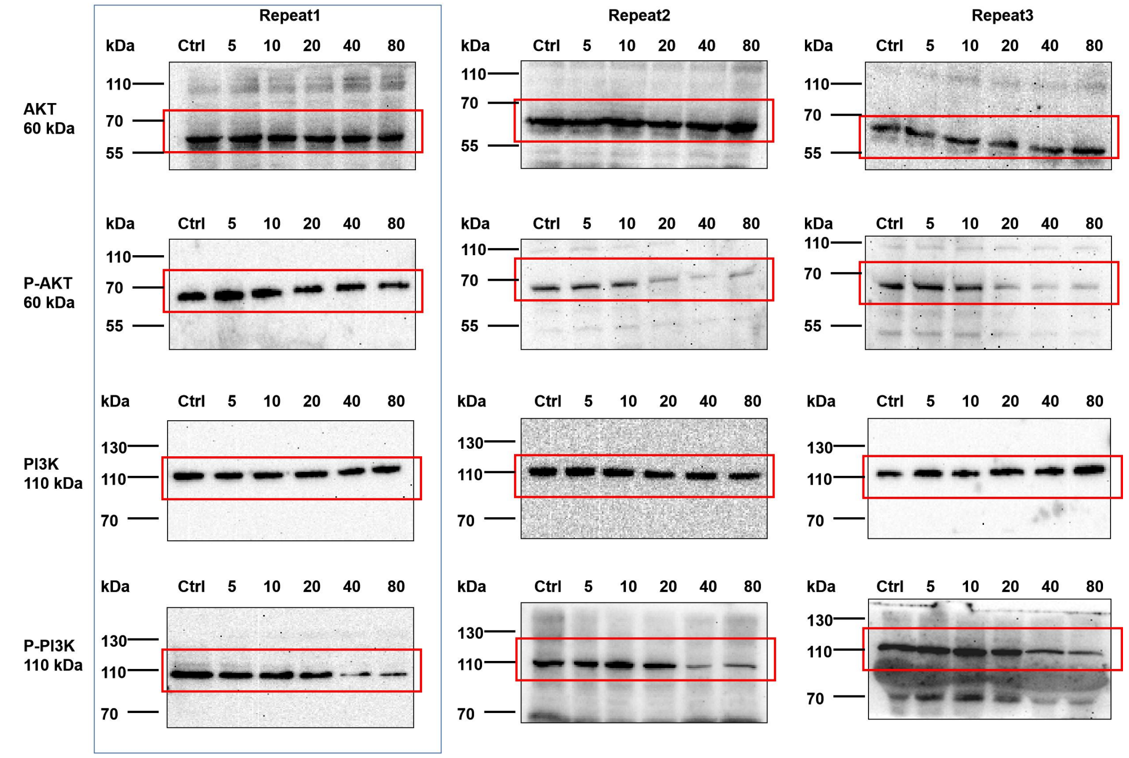


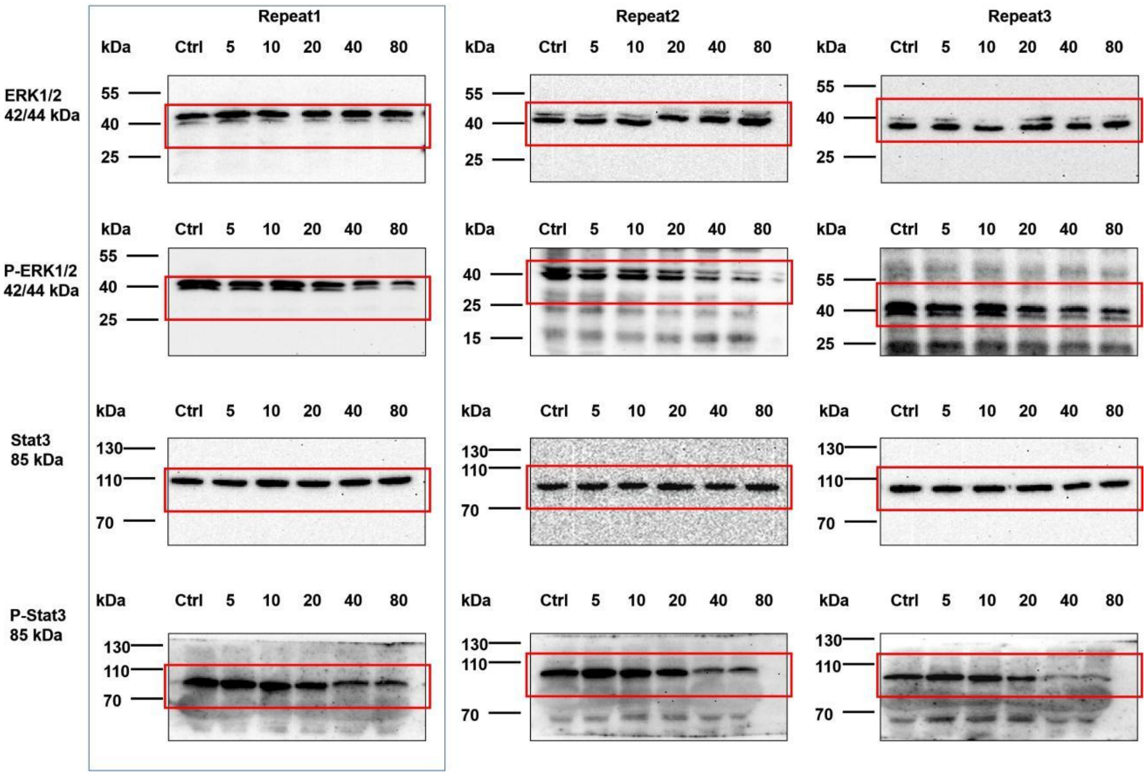

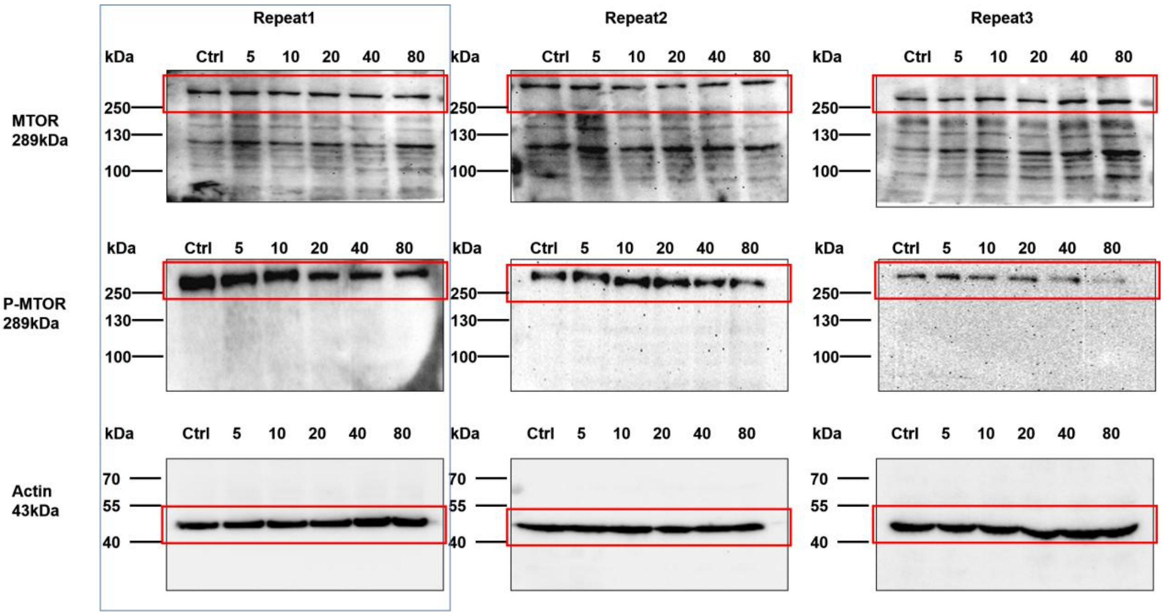


| | | | | | | | | | | | |
|---|---|---|---|---|---|---|---|---|---|---|---|
| Ctrl | 51144 | 1.565424995 | 1 | 51381 | 1.94213033 | 1 | 40621 | 1.622244409 | 1 | 1 | 0 |
| 5 | 44350 | 1.303989886 | 0.832994165 | 55702 | 2.021337591 | 1.0407837 | 45798 | 1.640270764 | 1.011111985 | 0.96162995 | 0.112385393 |
| 10 | 42300 | 1.284933171 | 0.820820656 | 47018 | 1.736262925 | 0.893999181 | 43715 | 1.467537263 | 0.904633886 | 0.873151241 | 0.045630492 |
| 20 | 40391 | 1.074657443 | 0.686495646 | 40283 | 1.43035188 | 0.736486042 | 34891 | 1.132677574 | 0.698216353 | 0.707066014 | 0.026143781 |
| 40 | 30940 | 0.867298312 | 0.554033771 | 26373 | 0.965478108 | 0.497123233 | 21993 | 0.80770502 | 0.497893545 | 0.516350183 | 0.032637217 |
| 80 | 29112 | 0.800461932 | 0.511338413 | 22983 | 0.836780019 | 0.430856779 | 20394 | 0.794097033 | 0.489505175 | 0.477233456 | 0.041620544 |

| | | | |
|---|---|---|---|
| Ctrl | 32671 | 26456 | 25040 |
| 5 | 34011 | 27557 | 27921 |
| 10 | 32920 | 27080 | 29788 |
| 20 | 37585 | 28163 | 30804 |
| 40 | 35674 | 27316 | 27229 |
| 80 | 36369 | 27466 | 25682 |

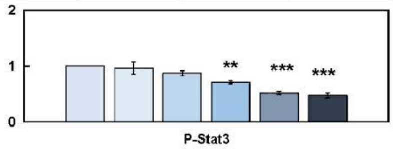


| | Repeat 1 | | | Repeat 2 | | | Repeat 3 | | | | |
|---|---|---|---|---|---|---|---|---|---|---|---|
| 6-SH (μM) | intden | Normalization to Actin (with same group) | Quadratic normalization to 0 (in each group) | intden | Normalization to Actin (with same group) | Quadratic normalization to 0 (in each group) | intden | Normalization to Actin (with same group) | Quadratic normalization to 0 (in each group) | The mean value of the three normalized groups | The standard deviation of the three normalized |
| Ctrl | 32536 | 1.049717696 | 1 | 34785 | 0.86882134 | 1 | 34053 | 0.797102128 | 1 | 1 | 0 |
| 5 | 32921 | 1.03848459 | 0.989298927 | 33806 | 0.770735489 | 0.887104693 | 30117 | 0.680271955 | 0.853431363 | 0.909944994 | 0.070754917 |
| 10 | 31049 | 0.951635149 | 0.906562928 | 28786 | 0.633703908 | 0.729383451 | 31604 | 0.765804841 | 0.960736165 | 0.865560848 | 0.121003709 |
| 20 | 26966 | 0.729047259 | 0.694517451 | 8384 | 0.187586701 | 0.215909407 | 15671 | 0.36481516 | 0.457676811 | 0.456034556 | 0.239308248 |
| 40 | 27727 | 0.773675986 | 0.737032432 | 7277 | 0.171744825 | 0.197675653 | 9625 | 0.231509321 | 0.290438719 | 0.408382268 | 0.288373793 |
| 80 | 23337 | 0.64370828 | 0.613220376 | 8973 | 0.206076891 | 0.237191332 | 3091 | 0.085238397 | 0.106935353 | 0.319115687 | 0.262896997 |

| | | | |
|---|---|---|---|
| Ctrl | 30995 | 40037 | 42721 |
| 5 | 31701 | 43862 | 44272 |
| 10 | 32627 | 45425 | 41269 |
| 20 | 36988 | 44694 | 42956 |
| 40 | 35838 | 42371 | 41575 |
| 80 | 36254 | 43542 | 36263 |

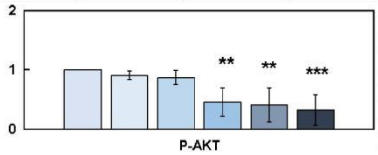


| | | | | | | | | | | | |
|---|---|---|---|---|---|---|---|---|---|---|---|
| Ctrl | 35904 | 1.127248752 | 1 | 59554 | 1.662729989 | 1 | 69919 | 2.014028114 | 1 | 1 | 0 |
| 5 | 36274 | 1.049260941 | 0.930815793 | 49154 | 1.571369202 | 0.945053744 | 52649 | 1.743921828 | 0.86588753 | 0.913919022 | 0.04220128 |
| 10 | 30409 | 0.954726696 | 0.846952986 | 41562 | 1.326376257 | 0.79770995 | 51799 | 1.681185291 | 0.834737747 | 0.826466894 | 0.025642238 |
| 20 | 29029 | 1.046731331 | 0.928571736 | 49111 | 1.488798618 | 0.895394098 | 45396 | 1.378183916 | 0.684292293 | 0.836086042 | 0.132499795 |
| 40 | 17072 | 0.597424412 | 0.529984541 | 38982 | 1.417011996 | 0.852220147 | 23215 | 0.854875534 | 0.424460576 | 0.602221755 | 0.222841253 |
| 80 | 11326 | 0.43237259 | 0.383564488 | 30484 | 0.984053199 | 0.591829826 | 23601 | 0.781024555 | 0.38779228 | 0.454395531 | 0.119040361 |

| | | | |
|---|---|---|---|
| Ctrl | 31851 | 35817 | 34716 |
| 5 | 34571 | 31281 | 30190 |
| 10 | 31851 | 31335 | 30811 |
| 20 | 27733 | 32987 | 32939 |
| 40 | 28576 | 27510 | 27156 |
| 80 | 26195 | 30978 | 30218 |

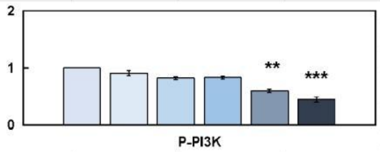


| | | | | | | | | | | | |
|---|---|---|---|---|---|---|---|---|---|---|---|
| Ctrl | 77866 | 2.498107154 | 1 | 73647 | 1.647547035 | 1 | 74571 | 1.969131238 | 1 | 1 | 0 |
| 5 | 65348 | 2.035256011 | 0.81471926 | 68161 | 1.510426131 | 0.916772692 | 72535 | 1.784510542 | 0.906242564 | 0.879244839 | 0.056128279 |
| 10 | 57490 | 1.980296924 | 0.792718967 | 62845 | 1.307772344 | 0.793769353 | 73178 | 1.949022532 | 0.989788032 | 0.858758784 | 0.113475872 |
| 20 | 53461 | 1.634593041 | 0.654332637 | 51912 | 1.072318275 | 0.650857458 | 55656 | 1.326500941 | 0.673647807 | 0.659612634 | 0.012278387 |
| 40 | 37281 | 1.19379423 | 0.477879513 | 36691 | 0.791897783 | 0.480652611 | 40160 | 0.963115737 | 0.489106931 | 0.482546352 | 0.005848369 |
| 80 | 24722 | 0.800738485 | 0.320538086 | 29536 | 0.608462775 | 0.369314357 | 17756 | 0.441296352 | 0.224107131 | 0.304653191 | 0.073895413 |

| | | | |
|---|---|---|---|
| Ctrl | 31170 | 44701 | 37870 |
| 5 | 32108 | 45127 | 40647 |
| 10 | 29031 | 48055 | 37546 |
| 20 | 32706 | 48411 | 41957 |
| 40 | 31229 | 46333 | 41698 |
| 80 | 30874 | 48542 | 40236 |

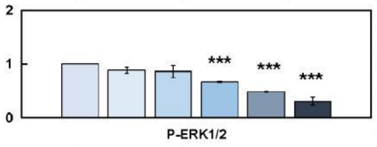

| | | | | | | | | | | | |
|---|---|---|---|---|---|---|---|---|---|---|---|
| Ctrl | 49714 | 1.251800373 | 1 | 34312 | 0.908806781 | 1 | 72067 | 1.569574213 | 1 | 1 | 0 |
| 5 | 46432 | 1.33463639 | 1.066173504 | 25365 | 0.823913467 | 0.90658816 | 68132 | 1.48277438 | 0.944698484 | 0.972486716 | 0.083342725 |
| 10 | 37934 | 1.36144708 | 1.087591209 | 23895 | 0.638767109 | 0.702863493 | 62774 | 1.468225938 | 0.935429447 | 0.90862805 | 0.193759103 |
| 20 | 31750 | 1.310034659 | 1.046520426 | 25421 | 0.810773745 | 0.892129947 | 52511 | 1.19156323 | 0.759163358 | 0.899271244 | 0.143811577 |
| 40 | 27031 | 1.057344025 | 0.84465866 | 20466 | 0.580875883 | 0.639163237 | 42696 | 0.990764376 | 0.631231303 | 0.705017733 | 0.120997604 |
| 80 | 23261 | 0.824419635 | 0.658587146 | 17014 | 0.447925442 | 0.492872029 | 37526 | 0.823353885 | 0.524571491 | 0.558676889 | 0.08796453 |
| Ctrl | 39714 | | | 37755 | | | 45915 | | | | |
| 5 | 34790 | | | 30786 | | | 45949 | | | | |
| 10 | 27863 | | | 37408 | | | 42755 | | | | |
| 20 | 24236 | | | 31354 | | | 44069 | | | | |
| 40 | 25565 | | | 35233 | | | 43094 | | | | |
| 80 | 28215 | | | 37984 | | | 45577 | | | | |

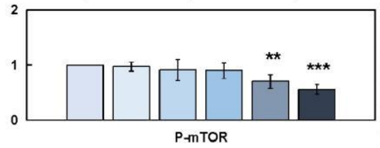


**Supplement Figure S4** Original unedited bands for evaluating 6-SH treatment (0-80 μM) affected the relevant signaling molecules in A549 cells by western blotting in Fig. 6A. Image software was used for sensitivity analysis, and the relative quantitative statistics and normalized the intensity value against “Ctrl” group were adopted. Western blotting was performed 3 times. The “Repeat 1” was used in the manuscript.
